# Thermal Transport Properties of Diamond Phonons by Electric Field

**DOI:** 10.3390/nano12193399

**Published:** 2022-09-28

**Authors:** Yongsheng Zhao, Fengyun Yan, Xue Liu, Hongfeng Ma, Zhenyu Zhang, Aisheng Jiao

**Affiliations:** 1 State Key Laboratory of Advanced Processing and Recycling of Nonferrous Metals, Lanzhou University of Technology, Lanzhou 730050, China; 2 School of Mechatronics Engineering, Lanzhou Institute of Technology, Lanzhou 730050, China; 3 Department of Safety Engineering, School of Petrochemical Engineering, Lanzhou University of Technology, Lanzhou 730050, China

**Keywords:** diamond, electric field, thermal transport

## Abstract

For the preparation of diamond heat sinks with ultra-high thermal conductivity by Chemical Vapor Deposition (CVD) technology, the influence of diamond growth direction and electric field on thermal conductivity is worth exploring. In this work, the phonon and thermal transport properties of diamond in three crystal orientation groups (<100>, <110>, and <111>) were investigated using first-principles calculations by electric field. The results show that the response of the diamond in the three-crystal orientation groups presented an obvious anisotropy under positive and negative electric fields. The electric field can break the symmetry of the diamond lattice, causing the electron density around the C atoms to be segregated with the direction of the electric field. Then the phonon spectrum and the thermodynamic properties of diamond were changed. At the same time, due to the coupling relationship between electrons and phonons, the electric field can affect the phonon group velocity, phonon mean free path, phonon–phonon interaction strength and phonon lifetime of the diamond. In the crystal orientation [111], when the electric field strength is ±0.004 a.u., the thermal conductivity is 2654 and 1283 $W\cdot m^{-1}K^{-1}$, respectively. The main reason for the change in the thermal conductivity of the diamond lattice caused by the electric field is that the electric field has an acceleration effect on the extranuclear electrons of the C atoms in the diamond. Due to the coupling relationship between the electrons and the phonons, the thermodynamic and phonon properties of the diamond change.

## 1. Introduction

With the continuous improvement in synthetic diamond technology, the application of diamond in quantum sensors, semiconductor components, and heat sinks of integrated circuits is becoming feasible. Generally, the band-gap of diamond is about 4.5 eV [1,2,3]. L. Wei et al. measured the thermal conductivity of diamond as exceeding 2000 $W\cdot m^{-1}K^{-1}$ by using the in-solid mirage effect thermal wave technique [4]. D.J. Twitchen et al. used the laser flash technique to measure the thermal conductivity of Chemical Vapor Deposition (CVD) diamond as high as 2200 $W\cdot m^{-1}K^{-1}\;$[5]. J. Hartmann et al. used photothermal microscopy to measure the local thermal conductivity of CVD diamond, and the results showed that the internal thermal conductivity of the CVD diamond was as high as 2200 $W\cdot m^{-1}K^{-1}\;$[6]. P. Chakraborty et al. used first-principles calculations to obtain a thermal conductivity of 2009 $W\cdot m^{-1}K^{-1}$ in the z direction of diamond at 0 GPa [7]. It can be seen that at room temperature, no matter which measurement method and calculation are used, the thermal conductivity of diamond is greater than 2000 $W\cdot m^{-1}K^{-1}\;$[8]. Diamond is a semiconductor material with wide-band gap, so diamond has excellent properties in terms of hardness. Diamond is also widely used in high-power Insulated Gate Bipolar Transistor IGBT equipment, power energy and other fields [9,10,11,12]. The excellent thermal conductivity of diamond is mainly due to its strong C−C bonds and light carbon atoms, resulting in a significantly higher phonon group velocity than other materials [13,14,15]. Furthermore, according to previous studies [16,17,18,19], the response of semiconductor materials to electric fields has practical application significance. The external electric field has a certain influence on the electronic wave function, electronic structure, lattice strain, atomic coordinates, dielectric constant, interatomic force constants, spectral properties, Born effective charge, and electrical conductivity of semiconductor materials. Therefore, it is feasible to control the thermal conductivity of diamond lattice by applying an external electric field.

In recent years, the response of materials to external electric fields through practical means and first-principles calculations has been widely studied. K. Kim et al. changed the alignment of titanium oxide (TiO) on the boron nitride (BN) surface by applying an external electric field when using titanium oxide (TiO) to modify the boron nitride (BN) surface form. At room temperature on disk samples using the laser flash method (LFA), the measured interfacial thermal conductivity increased from 0.78 to 1.54 $W\cdot m^{-1}K^{-1}$ [20]. W. H. Huber et al. applied a vertical or parallel electric field to SrTiO_3_ and KTaO_3_ single crystals. The thermal conductivity of the SrTiO_3_ was measured to increase by a factor of about 4 using a conventional steady-state heat flow technique [21]. Currently, the research on the response of diamonds under an electric field mainly focus on the changes in electronic and structural properties. For example, Y. Aikawa et al. found that diamond has the performance of a photoelectric switch when applying an electrostatic field to it; that is, diamond can keep working normally under an electrostatic field up to 2 × 10^6^ V∙cm^−1^. In addition, the carrier mobility and lifetime were measured, and it was found that diamond is sensitive to ultraviolet light under the electrostatic field [22]. K. S. Sankara Reddy et al. applied an electric field to a diamond-like film, and the diamond film was transformed into a more ordered structure [23]. The electronic properties of diamond nanowires under an external electric field were studied by Yanlin Gao et al. using density functional theory. They found that the electric field can lead to a high concentration of carriers at the edges [24]. According to N. Lambert et al., high-concentration B-doped diamond has a current multiplication effect under a high electric field [25]. The above studies have concluded from different aspects that the electric field has a certain influence on electronic and structural properties of diamond. However, there is no report on thermal transport response mechanism of diamond under an external electric field.

**Figure 1:**
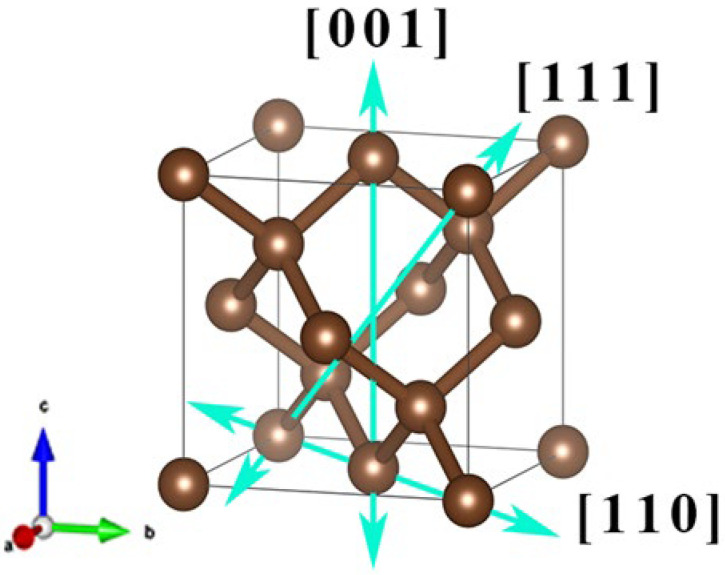
Schematic diagram of the diamond unit cell structure and the direction of the applied electric field, in which the solid line with the blue arrow is the direction of the applied electric field, and the crystal orientation indices corresponding to the applied electric field directions are:$\;\left[001\right]$, $\left[00\bar{1}\right]$, $\left[110\right]$, $\left[\bar{1}\bar{1}0\right]$, $\left[111\right]$ and $\left[\bar{1}\bar{1}\bar{1}\right]$, respectively.

In this work, the phonon and thermal transport properties of diamond are investigated using first-principles calculations by electric field. Specifically, positive and negative electric fields of different intensities were designed to be applied to the three crystallographic orientations of the original cell diamond. The equivalent diamond unit cell is shown in Figure 1. The lattice response of diamond primitive cells, the phonon dispersion relationship, and thermodynamic properties of diamond under an external electric field were explored. The lattice response of the properties of diamond phonon were investigated, including diamond thermal conductivity, phonon group velocity, phonon mean free path, phonon–phonon interaction strength and phonon lifetime density, to reveal thermal transport properties and response mechanism of diamond by external electric field. It is estimated that the electric field may regulate the thermal conductivity of the diamond lattice.

## 2. Methods and Models

### 2.1. Computational Details

In this work, the first-principles calculation method was used to explore the following contents, including phonon properties of a diamond under electric field, phonon dispersion relation, lattice thermal conductivity, interatomic force, phonon group velocity, phonon mean free path, phonon–phonon interaction strength and phonon lifetime. The phonon calculation software is the first-principles calculation software ABINIT [26,27,28,29,30] using Density-Functional Perturbation Theory (DFPT). The calculation of lattice thermal conductivity is based on ABINIT and Phono3py [31,32]. The crystal model, charge density and electron local function were visualized in 3D using VESTA [33] (Version: 3.1, Creator: Koichi Momma and Fujio Izumi, and Tsukuba-shi, Japan) software. The previous test results show that since Phono3py uses the finite difference method to calculate the thermal conductivity, the calculation of the lattice frequency is quite sensitive to the quality of the force. Therefore, when ABINIT calculates the response of the force, according to the test results, appropriately increasing the FFT box and the G-sphere ratio, the value is 4.5. Norm-conserving pseudopotentials (ONCVPSP) and Projector-augmented-wave (PAW) were used to describe the interactions between ionic real and valence electrons in this calculation, the Perdew–Burke–Ernzerhof (PBE) equation, using generalized gradient approximation (GGA) to control the exchange phase between electrons. The cutoff energy of the ONCVPSP pseudopotential is selected as 41 Ha. When PAW pseudopotential is used, the cutoff energy of coarse mesh and fine meshes are 15 Ha and 50 Ha, respectively. The cutoff energy for fine meshes is 50 Ha. Since electrons and phonons show different convergence rates, the convergence requirements for grid size are different [34,35], so the 12 × 12 × 12 grid is used to sample the Brillouin zone. In contrast, the q grid uses 56 × 56 × 56, the self-consistent loop iteration convergence precision is set to 1.0 d−12 Ha, and the corresponding atomic coordinate precision reaches the order of magnitude of 10 d−31. When Phono3py calculates the lattice thermal conductivity, the finite displacement method is used to calculate second-order force constants and third-order force constants. The second-order force constants and third−order force constants of diamond are calculated using supercells of 3 × 3 × 3 and 2 × 2 × 2, respectively. The atomic displacements are both 0.06 Å when the second-order force constant and the third-order force constant are calculated. The thermal conductivity of the diamond lattice is calculated by directly solving the Boltzmann phonon transport equations for the second and third force constants by the single-mode relaxation time approximation (RTA) method. The phonon group velocity, phonon mean free path, phonon–phonon interaction strength and phonon lifetime are calculated by taking the complete conductance calculation after the self-energy imaginary part of the lattice thermal conductivity. We have carefully checked the convergence of the computational parameters used in the first-principles calculations described above, based on [36] and our previous convergence test results.

For the response of diamond to an electric field, the ideal state is that two parallel electrode plates sandwich the bulk material. A uniform electric field is applied between the two parallel electrode plates, and the diamond’s response to different uniform electric fields is observed. In this work, we used the open source software ABINIT to calculate the interatomic force constant of diamond under an electric field, the total energy of the system, the change in the three-dimensional lattice structure, the electronic response and the phonon dispersion relationship. During the calculation, the basic procedure followed is to use the diamond structure after structural relaxation to perform the ground state calculation and then apply a uniform electric field according to the wave function calculated from the ground state. The electric field strength is gradually increased. Finally, the response of the diamond is calculated according to the database derived under the electric field (q-point dynamic matrix, dielectric tensor, Born effective charge, interatomic force constant, scattering potential). The method of directly applying a uniform electric field is adopted in the calculation process, compared with the calculation method that first calculates the structural response of the diamond primitive cell under the electric field. Then the lattice constant and atomic displacement parameters of the diamond primitive cell are extracted to construct the supercell to calculate the second-order force constant and the third-order force constant. The advantage of this calculation is the parameter consistency rate. Even better, the thermal transport response of diamond under an electric field can be quantitatively analyzed. In the preliminary verification stage, the diamond lattice thermal conductivity is calculated by the combination of ABINIT and Phonon3py. A small error with the experimental, numerical value reported in the relevant literature and the absolute error is less than 2%. They indicate that the selection of calculation parameters is more accurate, and the results are reliable. See Section 3.3 for details. 

According to the definition of crystal structure in solid-state physics, a diamond crystal belongs to the diamond structure, which is a crystal formed by two face-centered cubic lattices nested along the volume diagonal of the cubic unit cell offset by 1/4 unit. The structure belongs to the cubic crystal system in the Bravais lattice, and the space group number is Fd−3 m (No. 227), which is a complex space group. There is a quadruple helix axis parallel to the *z*-axis, with coordinates (1/2, 1/4) on the xy plane. Rotating 2π⁄4 and translating 1/4 of the unit cell side length along the axis is a symmetry operation, and the coordination number of each carbon atom is 4. Detailed information on the primary unit cell structure and atomic positions of the diamond is shown in Figure 1. For the <100> crystal orientation family of diamond, the [100], [010] and [001] crystallographic orientations are equivalent. The forward direction of the electric field is chosen here along the [001] crystallographic orientation. In the <110> crystal orientation family, the three crystal orientations in [110], [101] and [011] are equivalent directions, and the direction along the [110] crystal orientation is selected as the positive electric field facing the diagonal. On the <111> crystal orientation family, the space diagonal along the [111] crystal orientation is selected as the positive electric field. According to the research results of Park, M. and Irie, M. et al., when the electric field strength reaches 2 × 106 V∙cm^−1^ [37] and 1 × 107 V∙cm^−1^ [38], it can excite valence electrons to the conduction band in the intrinsic region by impact ionization events, which may further result in a cascade creation of electron–hole pairs. The values listed in Table 1 are the experimental data used to calculate the phonon dispersion curve of the diamond. It should be noted that when the electric field strength reaches 0.00736 a.u., the diamond structure becomes unstable, and the phonon spectrum produces an apparent imaginary frequency.

The overall calculation can be divided into three parts. The first part calculates the diamond phonon dispersion relationship and lattice thermal conductivity without an external electric field. The calculation results of this part will be used as the benchmark for later calculations to verify the rationality of the calculation parameters selected later. The second part is to fix the lattice parameters of the diamond primitive cell and to calculate the phonon dispersion relationship of diamond after applying electric fields of different directions and strengths. This part calculates a total of 6 electric field application directions, 8 electric field parameters and 48 sets of calculation data. The third part is to fix the lattice parameters of the diamond cell and apply electric fields of different strengths and directions. This part of the calculation is to study the response of the thermal transport of the diamond lattice to the electric field. Again, a total of 6 electric field application directions, 10 electric field parameters and 60 sets of calculation data are designed.

### 2.2. Electron−Phonon Matrix Elements

The e−ph matrix element $g_{mnv\left(k,\;q\right)}$ is:

(1) $$g_{mnv\left(k,q\right)=\langle \psi_{mk+q}\left|\Delta_{qv}V^{\mathrm{KS}}\right|\psi_{nk}}\rangle$$

where $\psi_{nk}$ is the KS Bloch state and $\Delta_{qv}V^{\mathrm{KS}}$ is the first-order variation of the self-consistent KS potential induced by the phonon mode *qv*. The scattering potential can be expressed as:

(2) $$\Delta_{qv}V^{\mathrm{KS}}\left(r\right)=e^{iq\cdot r}\Delta_{qv}v^{\mathrm{KS}}\left(r\right)$$

where $\Delta_{qv}v^{\mathrm{KS}}\left(r\right)$ is a lattice periodic function [39]. 

Our goal is to find an approximated solution to the linearized Boltzmann transport equation (BTE) [40] within the SERTA/MRTA approximation [39]. SERTA/MRTA are more accurate than the constant relaxation time approximation (CRTA) as the microscopic e−ph scattering mechanism is now included, thus leading to carrier lifetimes *τ* that depend on the band index n and the wavevector K. However, both SERTA and MRTA are still approximated solutions to the BTE and that a more rigorous approach would require to solve the BTE iteratively and the inclusion of many-body effects at different levels. For a review of the different possible approaches, see the review paper [41].

In the SERTA, the transport linewidth is given by the imaginary part of the electron−phonon (e−ph) self-energy evaluated at the KS energy [39]. The linewidth of the electron state due to the scattering with phonons is given by:

(3) $$\underset{\eta \to 0^{+}}{\lim}ℑ\left\{\sum \begin{array}{c} \mathrm{FM}\\ nk \end{array}\left(\varepsilon_{nk}\right)\right\}=\pi \underset{m,v}{\sum }\int_{\mathrm{BZ}}^{}\frac{dq}{\Omega_{\mathrm{BZ}}}{\left|g_{mnv}\left(k,q\right)\right|}^{2}\times \left[\begin{array}{c} \left(n_{qv}+f_{mk+q}\right)\delta \left(\varepsilon_{nk}-\varepsilon_{mk+q}+\omega_{qv}\right)\\ +\left(n_{qv}+1-f_{mk+q}\right)\delta \left(\varepsilon_{nk}-\varepsilon_{mk+q}-\omega_{qv}\right) \end{array}\right]$$

where *ν* is the phonon mode, *m* the final electron state (after the scattering), $n_{qv}\left(T\right)$ is the Bose−Einstein occupation number, $f_{mk+q}\left(T,\varepsilon_{F}\right)$ is the Fermi−Dirac occupation function, $\varepsilon_{nk}$ is the energy of the electron state and $\omega_{qv}$ is the phonon frequency for the phonon wavevector q. The integration is performed over the BZ for the phonon wavevectors and $g_{mnv}\left(k,q\right)$ is the e−ph matrix element. Only the Fan−Migdal (FM) part contributes to the linewidth as the Debye−Waller term is Hermitian.

In the SERTA, the transport lifetime $\tau_{nk}$ is inversely proportional to the e−ph self-energy linewidth:

(4) $$\frac{1}{\tau_{nk}}=2\underset{\eta \to 0^{+}}{\lim}ℑ\left\{\sum_{nk}^{\mathrm{FM}}\left(\varepsilon_{nk}\right)\right\}$$

In the MRTA, the back-scattering is included by expressing the transport lifetime as:

(5) $$\frac{1}{\tau_{nk}}=2\pi \underset{m,v}{\sum }\int_{\mathrm{BZ}}^{}\frac{dq}{\Omega_{\mathrm{BZ}}}{\left|g_{mnv}\left(k,q\right)\right|}^{2}\left(1-\frac{v_{nk\;\cdot \;v_{mk+q}}}{{\left|v_{nk}\right|}^{2}}\right)\times \left[\begin{array}{c} \left(n_{qv}+f_{mk+q}\right)\delta \left(\varepsilon_{nk}-\varepsilon_{mk+q}+\omega_{qv}\right)\\ +\left(n_{qv}+1-f_{mk+q}\right)\delta \left(\varepsilon_{nk}-\varepsilon_{mk+q}-\omega_{qv}\right) \end{array}\right]$$

where $v_{nk,\alpha }$ is the α-th cartesian component of the velocity operator:

(6) $$v_{nk}=\frac{\partial \varepsilon_{nk}}{\partial k}=\langle nk\left|\frac{\partial H}{\partial k}\right|nk\rangle$$

The generalized transport coefficients are defined by:

(7) $$L_{\alpha \beta }^{\left(m\right)}=-\int \frac{dk}{\Omega_{\mathrm{BZ}}}v_{nk},\alpha^{v}{}_{nk},\beta^{\tau }{}_{nk}{\left(\varepsilon_{nk}-\varepsilon_{F}\right)}^{m}\frac{\partial f}{\partial_{\varepsilon }}{|}_{\varepsilon_{nk}}$$

These quantities can be used to obtain different transport tensors such as the electrical conductivity σ, Peltier (Π) and Seebeck coefficients (S) and charge carrier contribution to the thermal conductivity tensors [42]. The electrical conductivity tensor, for instance, is given by:

(8) $$\sigma_{\alpha \beta }=\frac{1}{\Omega }L_{\alpha \beta }^{\left(0\right)}$$

Moreover, it can be divided into hole and electron contributions:

(9) $$\sigma =n_{e}u_{e}+n_{h}u_{h}$$

where $n_{e}$ and $n_{h}$ are the electron and hole concentrations in the conduction and valence bands, respectively, and μe and μh are the electron and hole mobilities, which can be obtained by selecting the conduction or valences states n in Equation (8).

For electrons, we have:

(10) $$n_{e}=\underset{n\in \mathrm{CB}}{\sum }\int \frac{dk}{\Omega_{\mathrm{BZ}}}f_{nk}$$

(11) $$\mu_{e}=\frac{1}{n_{e}\Omega }L_{n\in \mathrm{CB}}^{\left(0\right)}$$

where n∈CB denotes states in the conduction bands, and similar expressions hold for holes. At zero total carrier concentration, the Fermi level $\varepsilon_{F}$ is located inside the band gap so that $n_{e}=n_{h}$.

Using the single-mode relaxation-time approximation (RTA), the macroscopic thermal conductivity tensor $k_{latt}$ [31] is calculated as a sum of contributions from individual phonon modes *λ* according to:

(12) $$k_{latt}=\frac{1}{NV}\underset{\lambda }{\sum }k_{\lambda }=\underset{\lambda }{\sum }C_{\lambda }v_{\lambda }⊗v_{\lambda }\tau_{\lambda }$$

where $k_{\lambda }$ is the modal thermal conductivities, $C_{\lambda }$ is the (volumetric) heat capacities, $v_{\lambda }⊗v_{\lambda }$ is the tensor products of the group velocities and $\tau_{\lambda }$ is the lifetimes. *V* is the unit cell volume, and *N* is the number of phonon wavevectors (q) included in the summation. $C_{\lambda }$ and $v_{\lambda }$ are calculated within the harmonic approximation. $\tau_{\lambda }$ is calculated as the inverse of the phonon linewidths $\Gamma_{\lambda }$ as:

(13) $$\tau_{\lambda }=\frac{1}{2\Gamma_{\lambda }}$$

where $\Gamma_{\lambda }$ is calculated as a sum of three-phonon scattering processes (collision and decay events) whose probabilities are determined as the product of a three-phonon interaction strength $\emptyset_{\lambda \lambda^{\prime }\lambda^{\prime \prime }}$ and a set of conservation of energy terms:

(14) $$\Gamma_{\lambda }=\frac{18\pi }{\hbar^{2}}\underset{\lambda^{\prime }\lambda^{\prime \prime }}{\sum }{\left|\Phi_{-\lambda \lambda^{\prime }\lambda^{\prime \prime }}\right|}^{2}\times \left\{\begin{array}{c} \left(n_{\lambda^{\prime }}+n_{\lambda^{\prime \prime }}+1\right)\delta \left(\omega_{\lambda }-\omega_{\lambda^{\prime }}-\omega_{\lambda^{\prime \prime }}\right)\\ +\left(n_{\lambda^{\prime }}-n_{\lambda^{\prime \prime }}\right)\left[\delta \left(\omega_{\lambda }+\omega_{\lambda^{\prime }}-\omega_{\lambda^{\prime \prime }}\right)+\delta \left(\omega_{\lambda }-\omega_{\lambda^{\prime }}+\omega_{\lambda^{\prime \prime }}\right)\right] \end{array}\right\}$$

The phonon lifetime is calculated from the imaginary part of the phonon self-energy, which is calculated by Equation (14), and the self-energy imaginary part is calculated using the third-order coefficient of the anharmonicity (the lowest-order coefficient of the anharmonic part). Then, the phonon lifetime can be written as the reciprocal form of the phonon linewidth $2\Gamma_{\lambda }\omega_{\lambda }$, as follows:

(15) $$\tau_{\lambda }=\frac{1}{2\Gamma_{\lambda }\omega_{\lambda }}$$

where $\omega_{\lambda }$ is the phonon frequencies and $n_{\lambda }$ is the mode occupation numbers.

The averaged three-phonon interaction strength $P_{\lambda }$ is a useful quantitative measure of how strongly the phonons in a given material interact:

(16) $$P_{\lambda }=\frac{1}{{\left(3n_{a}\right)}^{2}}\underset{\lambda^{\prime }\lambda^{\prime \prime }}{\sum }{\left|\Phi_{\lambda \lambda^{\prime }\lambda^{\prime \prime }}\right|}^{2}$$

where $n_{a}$ is the number of atoms in the primitive cell, and there are thus $3n_{a}$ at each wavevector.

## 3. Results

### 3.1. Phonon Dispersion Relation of Diamond under Electric Field

This section mainly discusses the variation law of phonon dispersion relation of diamond under electric field. After fixing the lattice shape and atomic positions of the diamond primitive cell, the phonon spectrum changes caused by the breaking of diamond lattice symmetry and lattice deformation under the electric field are calculated.

The calculation of the phonon dispersion relation of diamond under an electric field is based on the “Berry phase” concept of modern polarization theory [43,44], so the calculated minimum total energy is divided into two parts. One part is the ground state energy of the system, and the other is the energy generated by the interaction between the external electric field and the ions and electrons in the system. In calculations with fixed lattice structures and atomic positions, only changes in the electron density distribution are considered. In this case, the symmetry of the lattice system will be reduced. Due to the asymmetric distribution of electrons under the electric field, the actual symmetry is lower than the lattice symmetry in the calculation under the electric field. The electric field here is an absolute electric field, not a simplified electric field, because the lattice structure and atomic positions are fixed during the calculation. By establishing the equation of motion of diamond, the energy and frequency of the “normal mode” of lattice vibration can be obtained. The phonon dispersion relation of a diamond includes six branches in total, and its acoustic phonon dispersion curve and optical phonon dispersion curve each have three branches. The acoustic branch is the synchronous motion of all the constituent atoms of the diamond at lower frequencies. The optical branch is its relative motion, and the frequency is higher. The frequency of the optical branch is higher than that of the acoustic branch, and the frequency of the longitudinal phonon branch is higher than that of the transverse phonon branch. Compared with other similar diamond-structured crystals, the degeneracy of the longitudinal and transverse optical branches at the Γ point in diamond is particularly special. In general, in crystals such as diamond-like sphalerite gallium nitride [45], BN [46], SiC [47] and sphalerite ZnO [48], due to the presence of two different kinds of atoms inside such crystals, the crystal structure exhibits anisotropy, which leads to the creation of polarity. The result is that in the process of longitudinal wave vibration, the two types of atoms will repeatedly generate and consume energy. However, since there is no such phenomenon in transverse waves, there is no energy change between the two types of atoms. The end result is therefore the separation of transverse and longitudinal waves in the phonon spectrum of diamond-like crystals. This phenomenon causes the transfer of energy, including thermal transport, to be very different in diamond and in crystals of diamond-like structure.

**Figure 2:**
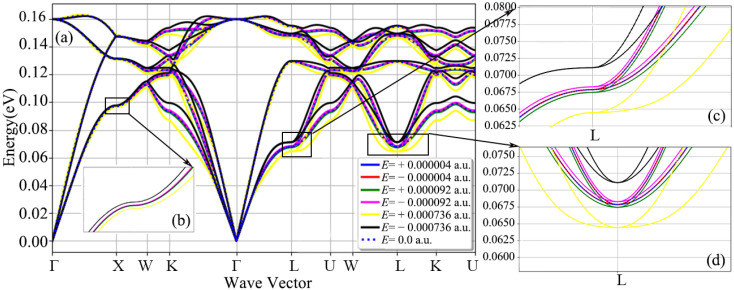
Phonon spectra of diamond primitive cells with fixed lattice structure and atomic positions after applying electric fields of different strengths in the [001] crystal direction: (**a**) full phonon dispersion along a high symmetry path, (**b**–**d**) are the phonon dispersion curve at point X and point L, respectively.

**Figure 3:**
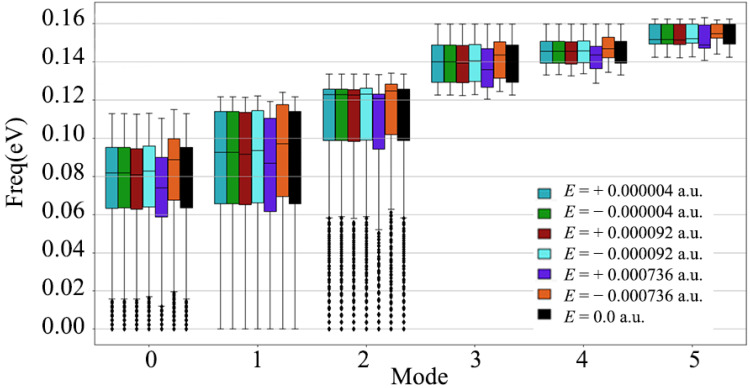
Frequency box plots of 3 acoustic branches and 3 optical branches of diamond, after applying different strength electric fields along the [001] crystal direction.

**Figure 4:**
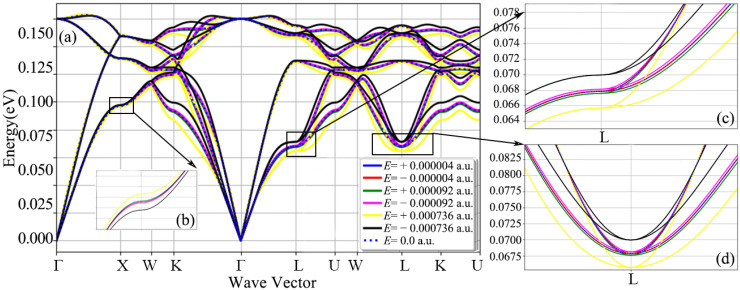
Phonon spectra of diamond primitive cells with fixed lattice structure and atomic positions after applying different electric fields in the [110] crystallographic direction: (**a**) full phonon dispersion diagram along a high symmetry path, (**b**–**d**) are the phonon dispersion relation curves at point X, point L, and point L, respectively.

**Figure 5:**
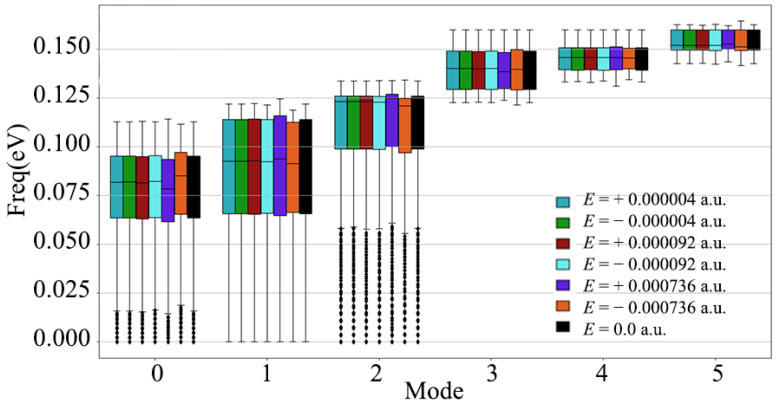
Frequency box plots of three acoustic branches and three optical branches of diamond after applying different electric field intensities along the [110] crystallographic direction.

**Figure 6:**
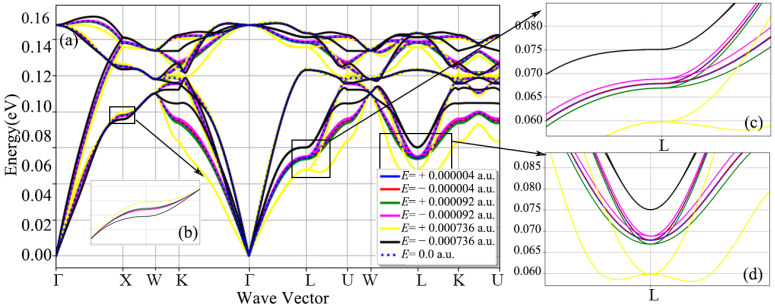
Phonon spectra of diamond primitive cells with fixed lattice structure and atomic positions after applying different electric fields in the [111] crystallographic direction: (**a**) full phonon dispersion diagram along a high symmetry path, (**b**–**d**) are the phonon dispersion relation curves at point X, point L and point L, respectively.

**Figure 7:**
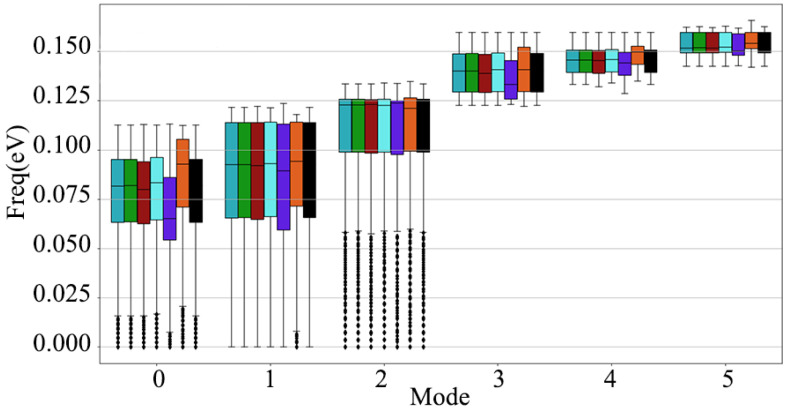
Frequency box plots of 3 acoustic branches and 3 optical branches of diamond, after applying different electric field intensities along the [111] crystallographic direction.

Figure 2, Figure 3, Figure 4, Figure 5, Figure 6 and Figure 7 are the diamond phonon spectra and frequency box plots under the application of electric fields of different directions and strengths. The abscissa in the phonon spectrum represents the path of the high symmetry point, and the ordinate represents the vibration frequency corresponding to the vibration modes in different wave vector directions. In the phonon spectrum, the blue dotted line represents the diamond phonon spectrum under a 0 a.u. electric field, which is in good agreement with the previous theoretical calculations or experimental results as the reference phonon spectrum [13,14,49,50,51]. Figure 2 is a graph of the full phonon dispersion along a highly symmetrical path after applying electric fields of different intensities in the diamond [001] crystallographic direction. In Figure 2, the electric field strengths are ±0.000004 a.u. and ±0.000019 a.u. Moreover, the diamond phonon spectrum undergoes only a slight numerical shift. When the electric field was increased to ±0.000736 a.u., the phonon spectrum shifted significantly. It can be seen from Figure 2a–c that the phonon curve shift caused by the application of positive and negative electric fields is basically symmetrical. For the three optical branches, the offset amplitude is relatively small, while for the three acoustic branches, especially at the L high symmetry point, the offset amplitude reaches ±0.0025 eV when the electric field strength is ±0.000736 a.u. The frequency box diagram of the diamond is shown in Figure 3. The three acoustic branches are more responsive to the electric field than the three optical branches. The acoustic branches of mode 0 to mode 3 are shifted to different degrees. The acoustic branch of mode 0 has the most significant cumulative frequency change of ±0.005 eV, when the electric field strength is ±0.000736 a.u. The mode 4 optical branch has the largest cumulative frequency change of ±0.003 eV, when the electric field strength is ±0.000736 a.u. The above results show that the low-frequency acoustic branch of diamond has a larger response than the high-frequency optical branch after applying an electric field in the [001] crystal direction.

Figure 4 shows a diagram of the total phonon dispersion relationship after applying electric fields of different intensities in the diamond [110] crystal orientation. In Figure 4, under the electric field of different strengths, the phonon spectrum is shifted to different degrees. From Figure 4b–d it can be concluded that when the electric field strength is ±0.000736 a.u., the corresponding offsets of the highly symmetrical X point, L point and L point are ±0.0015 eV, ±0.0021 eV and ±0.002 eV, respectively. 

In Figure 5, the response of the diamond’s three acoustic branches to the electric field varies more than the three optical branches. When the electric field strength is ±0.000736 a.u., Mode 1, Mode 2 and Mode 3 in the acoustic branch are shifted to different degrees, and the offsets are ±0.0017 eV, ±0.0018 eV and ±0.001 eV, respectively. Mode 4 has the largest offset among the three optical branches, and the offset is ±0.0008 eV. However, it should be noted that the offset of the acoustic branch of mode 1 under positive and negative electric fields is different from the lattice waves of other modes, and it is just opposite to the positive and negative response of the electric field. After positive and negative electric fields are applied to the crystal orientation of the diamond [110], the shift in the phonon spectra of modes 1, 2 and 4 are basically consistent, while the lattice waves 0, 3 and 5 are just opposite.

Figure 6 is a diagram of the total phonon dispersion of the diamond after applying different electric fields in the diamond [111] crystal orientation. In Figure 6, compared with the application of the electric field in the [001] and [110] crystallographic orientations, the shift in the diamond phonon spectrum is relatively strong, whether it is the phonon branch or the optical branch. At the high symmetry point L, as shown in Figure 6c, when an electric field of ±0.000736 a.u. is applied, the offset is ±0.0075 eV. On the whole, the offset of the three acoustic branches is larger than that of the three optical branches, especially the part far from the high symmetry point. In Figure 7, the frequencies of the three acoustic branches and the three optical branches are shifted to a certain extent after applying different electric fields in the diamond [111] crystal direction, and the acoustic branch of mode 0 is the most significant. When the applied electric field strength is ±0.000736 a.u., the frequency offset of the acoustic branch of mode 0 is at most 0.01 eV, while the offset of the optical branch of mode 3 is only 0.0021 eV. Special attention should be paid to the acoustic branch of mode 2. When the electric field strength is 0.000736 a.u., the acoustic branch of mode 1 has a significant drop in the Q1 quantile.

To sum up, under the action of the external electric field, the vibration mode of C atoms in a diamond will change, including the collective vibration and relative vibration of C atoms in the diamond unit cell. The main reason is that the electric field increases the kinetic energy of the free electrons in the C atoms and continuously accelerates the electrons. In fact, this acceleration is limited because the electrons are scattered. At the same time, due to the action of the periodic lattice field, the electrons are subjected to Bragg reflection at the boundary of the Brillouin zone. Therefore, the moving distance of the electrons under the action of the electric field is limited, but it is enough to change the vibration mode of the C atoms in the diamond unit cell. Essentially, changes in the harmonic force constants in the diamond unit cell due to the action of the electric field on the electrons lead directly to changes in the phonon dispersion relation. According to the analysis of the calculated results, when the electric field strength applied to the diamond exceeds ±0.000736, the imaginary frequency in the phonon spectrum will appear, and the acoustic branch will demonstrate serious tailing deformation. This phenomenon is due to the crystal caused by severe damage to lattice symmetry.

### 3.2. Charge Density Characteristics of Diamond under the Action of External Electric Field

**Figure 8:**
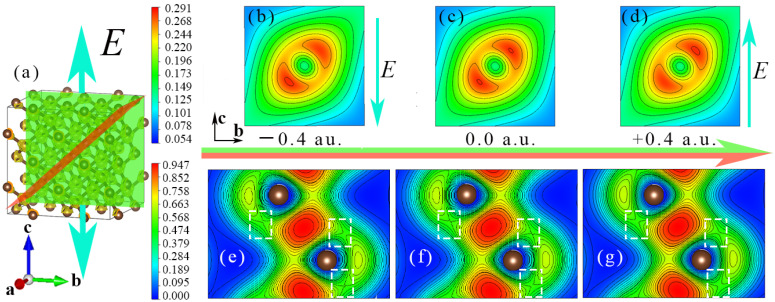
Charge density and electron localization function (ELF) of diamond under external electric field. (**a**) The picture shows the diamond 2 × 2 × 2 supercell model and the schematic diagram of the electric field direction, in which the grass green section and the orange section are the 2D charge density section and the 2D electron local function section, respectively. The (**b**–**d**) plots represent the C atomic charge densities at electric field strengths of −0.4 a.u., 0.0 a.u. and +0.4 a.u., respectively. The (**e**–**g**) show the charge transfer around C−C bonds at electric field strengths of −0.4 a.u., 0.0 a.u. and +0.4 a.u., respectively.

In Figure 8a, the external electric field is applied along the diamond [001] crystallographic direction. The tangential direction of the 2D charge density is [100], 6.393 Å from the origin. The tangent direction of the electron local function is the [$\left[0\bar{1}1\right]$] direction, and the distance from the origin is 1.264 Å. It can be seen from Figure 8b–d that the electric field exerts a force on the electrons around the C atom, resulting in a shift in the electron density outside the nucleus of the C atom, and the direction of the force is consistent with the direction of the electric field. In particular, the closer it is to the C nucleus, the greater the force. The main reason for this is that the closer the distance to the C nucleus, the greater the electron density, so the force of the external electric field on the electrons is more concentrated. For Figure 8e–g, the influence of the external electric field on the C−C bond is mainly investigated. For the application of electric field along the [001], [110] and [111] crystallographic directions, the C−C bond length does not change, and the C−C bond length is 1.547 Å with or without the electric field. However, according to the calculation results of the diamond electron local function; although the external electric field does not obviously change the covalent bond between C atoms, it changes the distribution of electrons around the C−C bond. In the white dashed box in Figure 8f, in the absence of an external electric field, the distribution of electrons around the C−C bond presents a symmetrical distribution, and a typical single C atom forms a covalent bond with four surrounding C atoms. However, under the action of an electric field with a strength of −0.4 a.u., the electrons around the C−C bond obviously move down along the direction of the electric field. In Figure 8e, the circled by the white dashed box in the lower right corner, compared with the circled by the white dashed box in the lower right corner of Figure 8f, the local electrons are obviously extended. In Figure 8e,f, circled by the white dotted box in the middle position, under the action of negative electric field, the local electrons shrink. Under the action of a +0.4 a.u. electric field, as shown in Figure 8g, the effect is just opposite to that of negative electric field.

### 3.3. Thermodynamic Properties of Diamond under the Action of Electric Field

**Figure 9:**
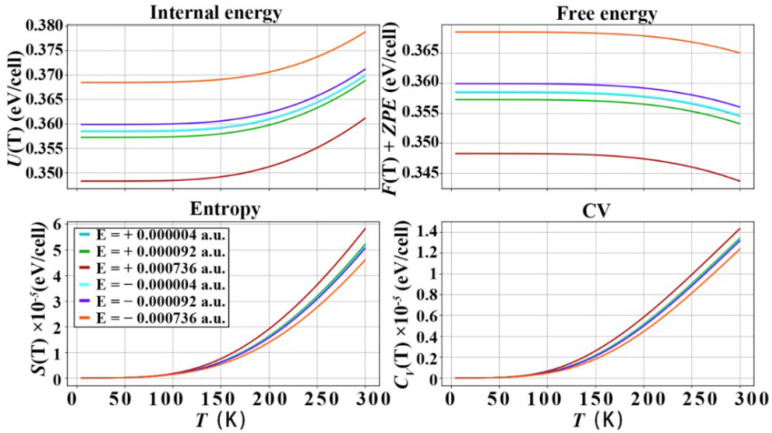
Thermodynamic properties of diamond, after applying different electric fields in the [001] crystallographic direction of diamond.

**Figure 10:**
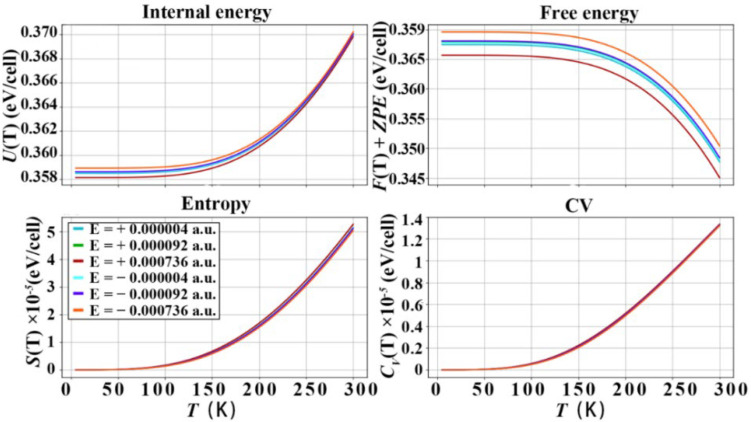
Thermodynamic properties of diamond, after applying different electric fields in the [110] crystallographic direction of diamond.

**Figure 11:**
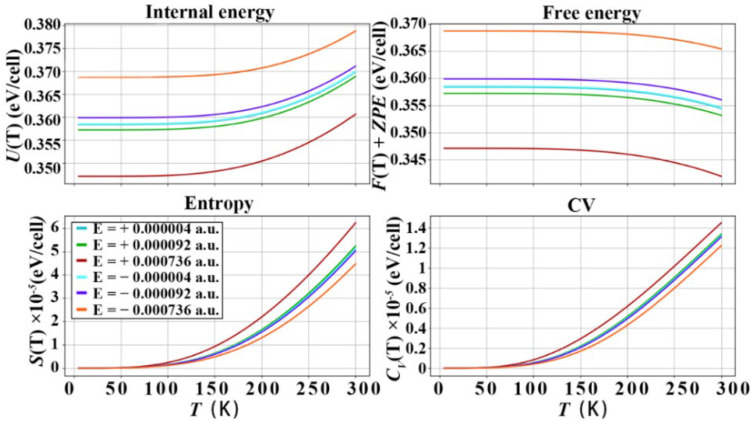
Thermodynamic properties of diamond, after applying different electric fields in the [111] crystallographic direction of diamond.

The diamond e–ph matrix elements under the action of electric field are calculated by Equations (1) and (2), and the imaginary part of the phonon self-energy is calculated, and the thermodynamic properties are calculated and plotted in the harmonic approximation as shown in Figure 9, Figure 10 and Figure 11, in the temperature range of 0−300 K, where *U*(T) is the internal energy, *F*(T)+*ZPE* (zero-point free energy) is the free energy, *S*(T) is the entropy and *C*_V_ is the constant volume specific heat capacity. It should be noted that the energy unit of the above calculation is eV, and the thermodynamic quantity is calculated based on the lattice unit. The *U*(T) and *F*(T)+*ZPE* of diamond under the action of forward electric field are reduced to different degrees. The *S*(T) and *C*_V_ of diamond are improved under the action of the forward electric field. In particular, the electric field effect of the [111] crystal orientation is the most obvious. The *U*(T) decreased to 0.361 eV/cell, then *F*(T)+*ZPE* decreased to 0.342 eV/cell, the *S*(T) increased to 6 × 10^−5^ eV/cell, and the *C*_V_ increased to 1.5 × 10^−4^ eV/cell. Under the action of a negative electric field. The *U*(T) and *F*(T)+*ZPE* are increased to 0.379 eV/cell and 0.365 eV/cell, respectively. The *S*(T) and *C*_V_ drop to 4.2 × 10^−5^ eV/cell and 1.25 × 10^−4^ eV/cell, respectively, at 300 K. The effect of the negative electric field is just the opposite of that of the positive electric field, and the *U*(T) and *F*(T)+*ZPE* of diamond are increased to varying degrees. The *S*(T) and *C*_V_ of diamond are reduced under the action of the positive electric field. However, the overall improvement or reduction is not as obvious as that of the positive electric field. In the [001] and [110] crystallographic orientations, the effect of positive and negative electric fields is basically the same as that of the [111] crystallographic orientation, but the effect is slightly weaker. From the above analysis, the following conclusions can be drawn: Applying a positive electric field to the diamond will increase the energy in the diamond unit cell. The reason is that the electric field interacts with the free electrons of the diamond, increasing the chaos of the microscopic particles inside the diamond. It is the acceleration of free electrons and the increased scattering of free electrons caused by the electric field which reduces the *U*(T) and *F*(T)+*ZPE* of the diamond unit cell. However, on the contrary, due to the increase in the disorder of the microscopic particles inside the diamond, the potential energy and kinetic energy of the microscopic particles in the diamond unit cell increase, that is, the thermal energy that the C atoms in the diamond can carry increases, increasing the specific heat of constant volume. According to Equation (12), the increase in the diamond’s constant volume specific heat capacity will increase the lattice’s thermal conductivity. Therefore, applying a positive electric field to diamond will improve its thermal conductivity.

### 3.4. Variation in Lattice Thermal Conductivity of Diamond under Electric Field

As described in Section 2.1, the calculation parameters used to calculate the thermal conductivity of the diamond lattice is shown in Table 2.

**Figure 12:**
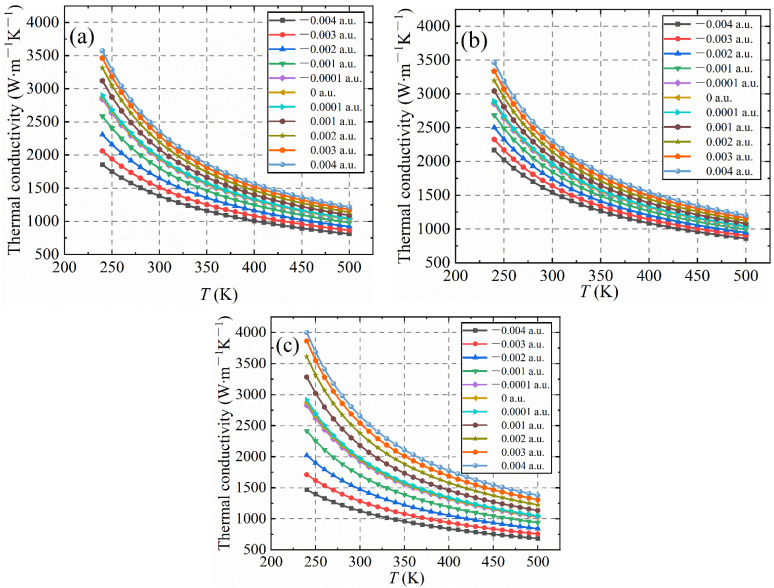
Variation curves of lattice thermal conductivity of diamond in the [001], [110] and [111] crystallographic directions after positive and negative electric fields are applied (temperature range is 240−500 K). (**a**) The positive and negative electric fields are applied to the [001] crystallographic direction; (**b**) the positive and negative electric fields are applied to the [110] crystallographic direction; and (**c**) the positive and negative electric fields are applied to the [111] crystallographic direction.

The change in the thermal conductivity of diamond with or without an electric field is shown in Figure 12. Figure 12a–c represent the thermal conductivities of the diamond after applying electric fields of different strengths and directions in the [001], [110] and [111] crystallographic directions, respectively. Temperatures range from 240 K to 500 K. When calculating the thermal conductivity of diamond, since the pseudopotential used is PAW, as the projectors depend on ion location, an additional force and additional stress terms arise. Therefore, the thermal conductivity of the diamond lattice will change quantitatively. According to the calculation benchmark of the thermal conductivity of diamond, the thermal conductivity of the diamond lattice under 0 a.u. is 1960.80 $W\cdot m^{-1}K^{-1}$. Compared with the diamond lattice’s thermal conductivity of 2000 $W\cdot m^{-1}K^{-1}$ reported in other literature [4,5,7,8,50], the absolute error is less than 2%. In this part of the calculation process, the increment in the electric field strength is 0.001 a.u. The rate of change of the thermal conductivity of the diamond lattice is $\Delta_{i}=TC_{i+1}-TC_{i}$. When the temperature is 300 K, the thermal conductivity change rates after applying different electric fields in the diamond’s three-crystal direction are shown in Table 3. 

As can be seen from Figure 12a and Table 3, After a positive electric field is applied to the [001] crystal direction of the diamond, the thermal conductivity of the diamond lattice increases gradually with the increase of electric field intensity, but $\Delta_{i}$ decreases with the increase in electric field intensity. On the contrary, when a negative electric field is applied, the thermal conductivity of the diamond lattice gradually decreases. Although −$\Delta_{i}$ also decreases with the decrease in the negative electric field strength, the change rate of $\Delta_{i}$ under the action of the negative electric field is significantly larger than that of the positive electric field. As can be seen from Figure 12b and Table 3, after a positive electric field is applied to the diamond [110] crystal direction, the lattice thermal conductivity of the diamond will also increase with the increase in the applied electric field strength. However, the maximum value of $\Delta_{i}$ is only 98.08 $W\cdot m^{-1}K^{-1}$, which is significantly smaller than the maximum value of $\Delta_{i}$ of 133.00 $W\cdot m^{-1}K^{-1}$ when a positive electric field is applied in the [001] crystal direction. Similarly, applying a negative electric field to the [110] crystallographic orientation will also decrease the diamond’s thermal conductivity. When the electric field strength decreases from 0 a.u. to −0.003 a.u., the change of $-\Delta_{i}$ is basically the same. When the electric field strength decreases to −0.004 a.u., $-\Delta_{i}$ decreases to −95.86 $W\cdot m^{-1}K^{-1}$. For the application of an electric field in the diamond [111] crystallographic direction, no matter whether the applied electric field is a positive electric field or a negative electric field, compared with the [001] and [110] crystal orientations, the rate of change of the thermal conductivity of the diamond lattice is pronounced. When a positive electric field is applied, the maximum value of $\Delta_{i}$ is 228.87 $W\cdot m^{-1}K^{-1}$, and up to 0.004 a.u., the value of $\Delta_{i}$ is greater than 100 $W\cdot m^{-1}K^{-1}$. For the negative electric field applied, the maximum value of $-\Delta_{i}$ is −249.92 $W\cdot m^{-1}K^{-1}$, and the negative electric field strength reaches −0.004 a.u., the values of $-\Delta_{i}$ are all less than −150 $W\cdot m^{-1}K^{-1}$. The above calculation results show that in the electric field, the thermal conductivity change rates of the three crystal directions of diamond show anisotropy, and the responses to positive and negative electric fields are just the opposite. For the positive electric field applied to the diamond’s three axial crystallographic directions, the diamond lattice’s thermal conductivity will be improved. The thermal conductivity change rate of the [111] crystal orientation is the largest, followed by the [001] crystal orientation and the lowest thermal conductivity change rate of the [110] crystal orientation. For the negative electric field applied to the diamond’s three crystallographic directions, the diamond lattice’s thermal conductivity will be reduced. The rate of change of the thermal conductivity of the diamond lattice is greater than that of the positive electric field, and the [111] crystallographic orientation is the largest, followed by the [001] crystallographic orientation, and the [110] crystallographic orientation is the smallest. 

**Figure 13:**
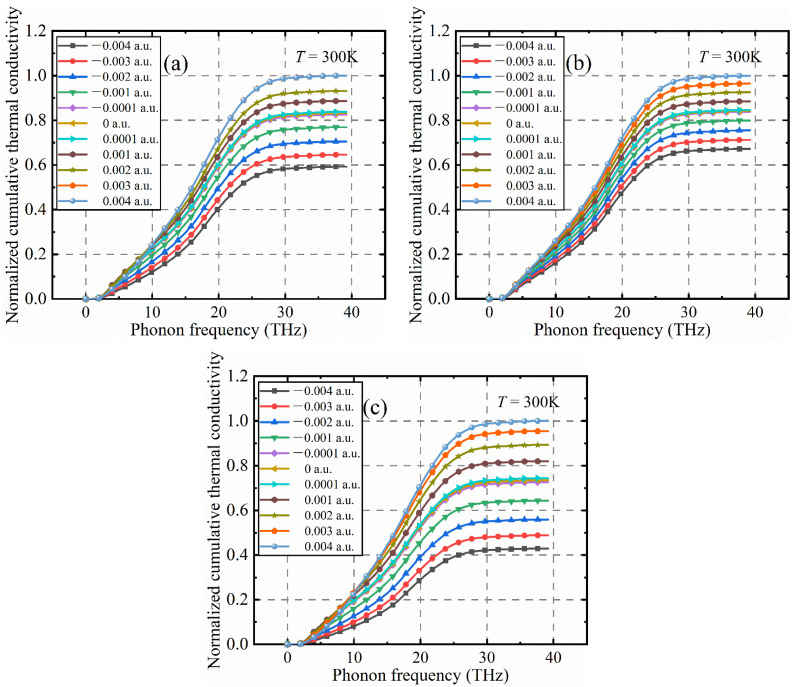
Frequency-normalized cumulative lattice thermal conductivity of diamond phonon: (**a**) positive and negative electric fields are applied to the [001] orientation, (**b**) positive and negative electric fields are applied to the [110] orientation and (**c**) positive and negative electric fields are applied to the [111] orientation. (T = 300 K).

Figure 13a–c show the calculated relationships between the normalized diamond phonon frequency and the cumulative lattice thermal conductivity after applying electric fields of different intensities in the three crystallographic directions of the diamond. It can be clearly seen that the relationship between the thermal conductivity of the lattice and the strength of the electric field applied to the diamond axis is basically consistent with the results in Figure 11. In Figure 13, the contribution to the thermal conductivity of the diamond lattice is mainly concentrated in the low-frequency phonon part, that is, the contribution of the phonon with frequency less than 30 THz, and the phonon branch with a frequency greater than 30 THz is relatively flat. On the other hand, phonons between frequencies of 0 and 30 THz and phonon branches between 10 and 25 THz contribute relatively more to the lattice’s thermal conductivity. 

**Figure 14:**
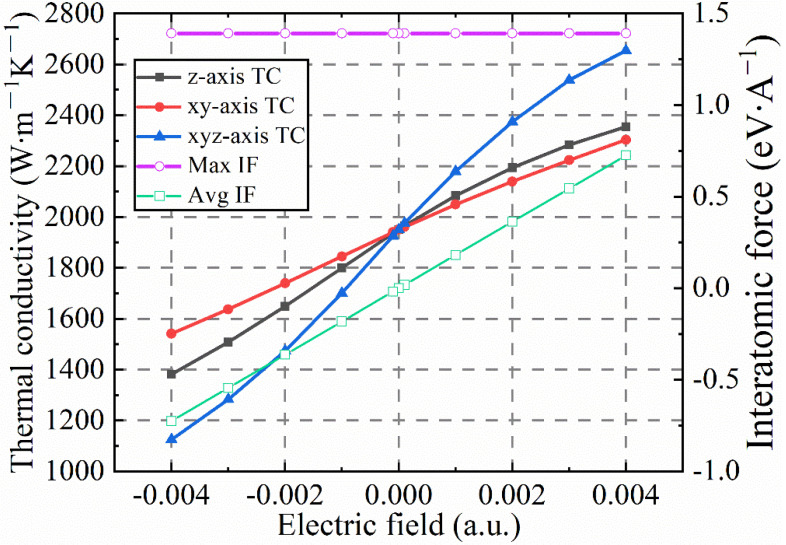
The relationship between lattice thermal conductivity, maximum force between atoms, average force and electric field intensity after applying an electric field in three crystallographic directions of diamond (T = 300 K).

In order to further explore the reasons why the positive and negative electric fields lead to changes in the thermal conductivity of diamond, the relationships among the lattice thermal conductivity, the maximum interatomic force, the average interatomic force and the electric field strength are shown in Figure 14. The lavender solid line represents the maximum interatomic force, and the turquoise solid line represents the average interatomic force. It should be noted here that the lavender solid line and the turquoise solid line represent the maximum interatomic force and the interatomic average force after positive and negative electric fields are applied to the three crystallographic directions of the diamond. In Figure 14, under the action of positive and negative electric fields in the three crystal directions of diamond, there is no significant change in the maximum force between diamond atoms, but the average force between atoms is proportional to the change in the electric field. In short, the average force between diamond atoms increases linearly with the increase in the applied electric field strength, and the rate of change in the three crystallographic directions is basically the same. This is inconsistent with the change law of the lattice thermal conductivity of the three crystal directions of diamond under the action of the electric field, which indicates that other factors respond to the electric field and affect the lattice thermal conductivity of the diamond. To this end, the key factors affecting the lattice thermal conductivity, phonon group velocity, phonon mean free path, phonon–phonon interaction strength and phonon lifetime are calculated.

### 3.5. Phonon Thermal Transport Properties of Diamond under Electric Field

**Figure 15:**
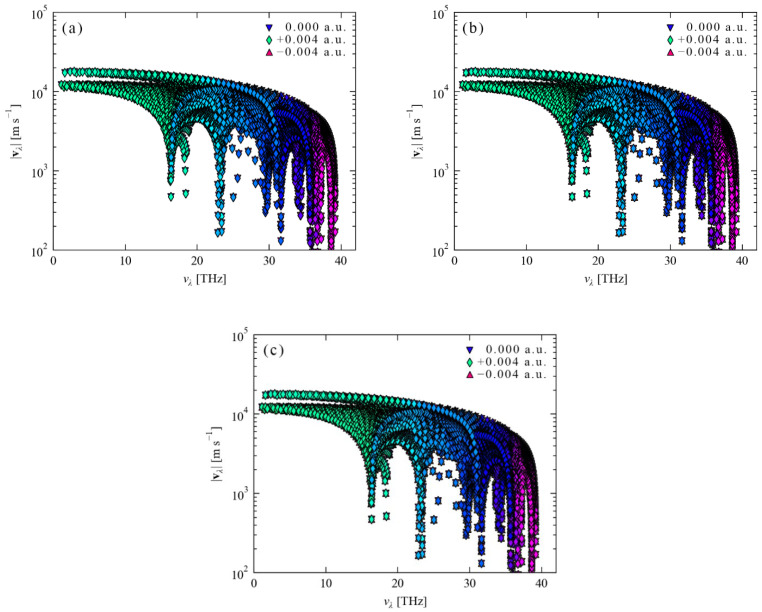
Phonon group velocities after applying ±0.004 a.u. electric field in diamond [001], [110] and [111] crystallographic orientations. Among them, (**a**) an electric field is applied in the [001] crystallographic direction, (**b**) an electric field is applied in the [110] crystallographic direction and (**c**) an electric field is applied in the [111] crystallographic direction.

**Figure 16:**
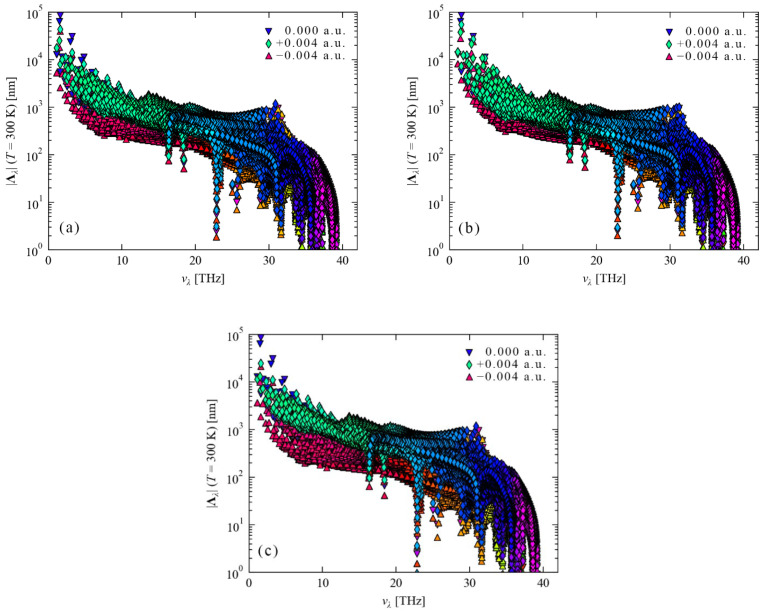
Phonon mean free path after applying ±0.004 a.u. electric field in diamond [001], [110] and [111] crystallographic orientations. An electric field is applied in the [001] crystallographic direction (**a**), an electric field is applied in the [110] crystallographic direction (**b**) and an electric field is applied in the [111] crystallographic direction (**c**).

As can be seen from Figure 15a–c, the positive and negative electric fields applied to a diamond in the [001], [110] and [111] crystallographic directions have little effect on the diamond’s phonon group velocity. After the diamond lattice vibration is quantized, the energy of its simple harmonic vibration is $\hbar \omega \left(q\right)$, and this energy is called a phonon. The phonon velocity of diamond, that is, the velocity of the simple harmonic vibration of C atoms, is only related to the density of C atoms in diamond. However, the external electric field cannot change the density of C atoms in the diamond, so the phonon velocity of the diamond does not respond to the external electric field. For diamonds, the phonon velocities of different frequencies are not nearly the same. In particular, the velocity of the low-frequency phonon group is relatively high, with a maximum value of 1 × 10^4^ m/s. As the frequency increases, the phonon group velocity decreases to varying degrees. In Figure 16a–c, the mean free paths of phonons after applying positive and negative electric fields in the [001], [110] and [111] crystallographic directions of the diamond unit cell. In the absence of an external electric field, as the phonon frequency increases, the mean free path of phonons gradually decreases from 1 × 10^5^ nm to about 1 × 10^2^ nm. It can be seen that the low frequency phonons show a more “brisk” state, and therefore, the mean free path is larger. The high-frequency phonons show the characteristics of “heavy and slow”, so the mean free path is small. For the contribution of the phonon mean free path to the lattice thermal conductivity, the longer the phonon free path, the better the energy transfer. It can be seen that the mean free path of phonons with frequencies less than 30 THz in diamond is basically greater than 1 × 10^2^ nm. Therefore, this part of the phonons contributes more to the lattice thermal conductivity. After the external electric field is applied, the phonons’ mean free path of is shifted significantly, especially the “brisk” phonons with smaller frequency shifts. In Figure 16, the blue inverted triangle, the green diamond and the orange-red right triangle represent the phonon mean free paths under the electric fields of 0 a.u, +0.004 a.u. and −0.004 a.u., respectively. After applying an electric field of +0.004 a.u. in the [001], [110] and [111] diamond cell orientations, the mean free paths of phonons in the three crystallographic frequency range between 0 and 30 THz all increased. Especially the mean free path of phonons at 0−17 THz in Figure 16c ([111] orientation), the overall broadening narrows and moves upward. Figure 16a ([001] crystal orientation) also shows a similar situation, but the degree of narrowing is smaller. Figure 16b ([110] crystal orientation) only shows the phenomenon of upward translation. However, when an electric field of −0.004 a.u. is applied to the crystal orientations of the diamond unit cells [001], [110] and [111], the phonon free path changes are dominated by downward translation, mainly concentrated on phonons with frequencies between 0 and 30 THz. However, the amount of downward translation is the largest in the crystal orientation of Figure 16c [111], followed by the crystal orientation of [001] in Figure 16a, and the smallest in the crystal orientation of Figure 16b [110]. 

**Figure 17:**
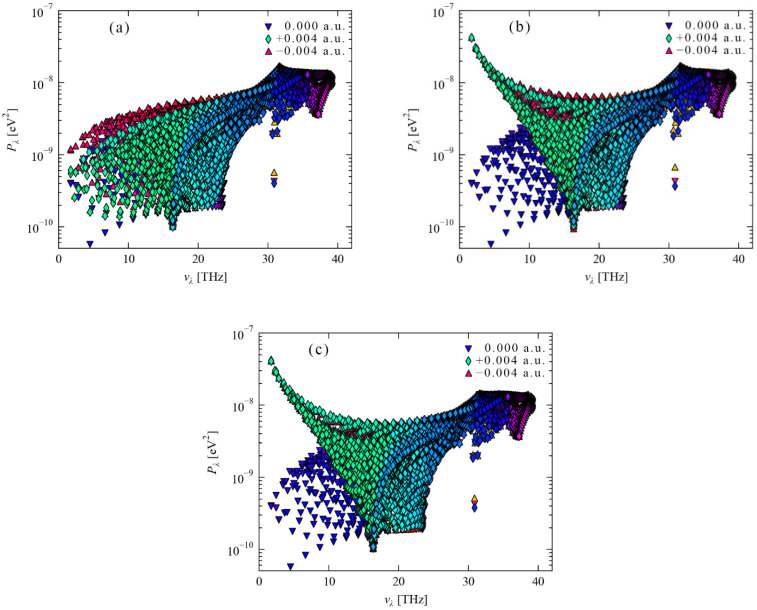
Phonon–phonon interaction strength after applying ±0.004 a.u. electric fields in the [001], [110], and [111] crystallographic orientations of the diamond unit cell. Among them, (**a**) an electric field is applied in the [001] crystallographic direction, (**b**) an electric field is applied in the [110] crystallographic direction and (**c**) an electric field is applied in the [111] crystallographic direction.

The phonon–phonon interaction strengths were calculated using the linear tetrahedron method for the three crystallographic orientations of the diamond. The phonon–phonon interaction strength results of the three crystallographic directions of diamond are shown in Figure 17a–c, respectively. After the electric field is applied to the three crystallographic directions, the diamonds all show a frequency of 30 THz as the demarcation point. Under the action of a positive electric field, the phonon–phonon interaction strength greater than 30 THz decreases, and the phonon–phonon interaction strength less than 30 THz increases, which is somewhat similar to the “lever” phenomenon, and 30 THz is just the fulcrum. Under the negative electric field, the phonon–phonon interaction strength shows the opposite phenomenon. The phonon–phonon interaction strength changes in diamond [110] and [111] crystal orientations after positive and negative electric fields are applied basically the same. Although the frequency of 30 THz is still used as the dividing point, the phonon–phonon interaction strength of less than 16 THz shows an almost exponential change, indicating that the phonon–phonon interaction in diamond is more sensitive to the electric field in the [110] crystallographic direction. A hole region appears between the frequencies of 8 THz and 18 THz for the crystal orientations of diamond [110] and [111] under the action of a positive electric field. Furthermore, after applying a negative electric field in the [111] orientation, the top phonon interaction strength between 10 THz and 25 THz is higher than that in the [110] orientation. To sum up, the phonon is a quantized treatment of the diamond lattice vibration, that is, the decoupling of the lattice wave. However, in practice, there is an energy exchange between lattice waves of various frequencies, and the phonon-phonon interaction strength is used to describe this exchange of energy. As a form of energy transfer, lattice wave belongs to the state of motion in three-dimensional space. As shown in Figure 17a, after applying the electric field in the crystal direction [001], only the amplitude direction of the phonon–phonon interaction strength of the simple harmonic vibration is enhanced, and it has a certain directionality. After applying an electric field in the [110] crystal direction, the transfer direction of the simple harmonic vibration is enhanced, which will promote the improvement of the thermal conductivity of the lattice. Variations occur in phonon–phonon interaction strength after an electric field is applied to the [111] crystallographic direction. The effect of the external electric field on the phonon is mainly concentrated in the propagation direction of the phonon, not the vertical direction.

**Figure 18:**
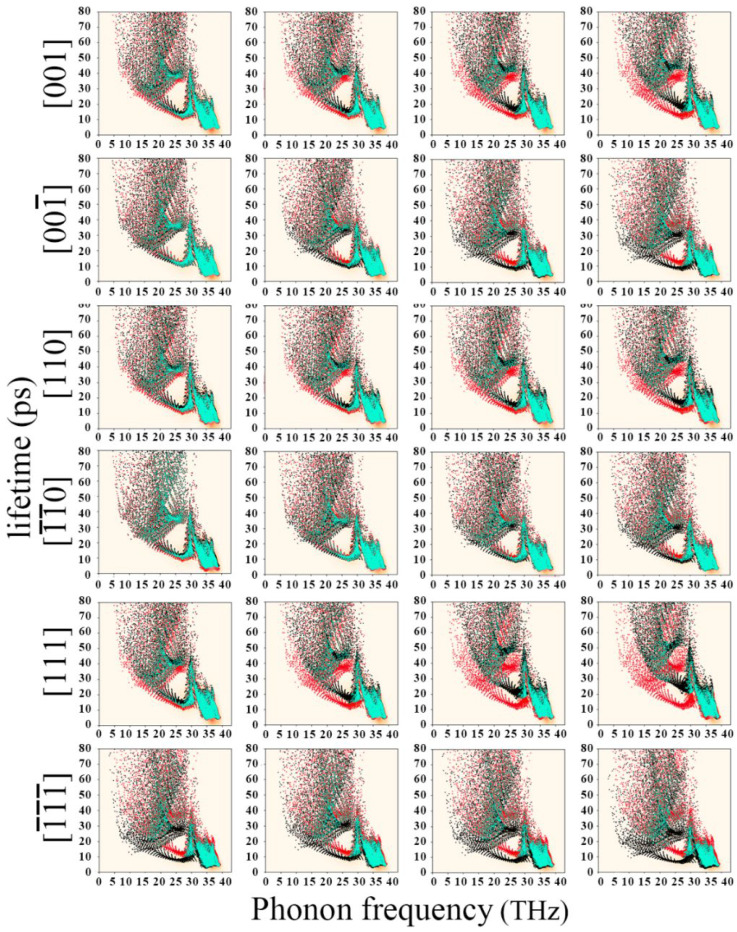
Plot of phonon lifetime versus frequency after the electric field is applied in the diamond’s 3 crystallographic orientations.

Figure 18 show the phonon lifetime density plots after applying different electric fields in the [001], [110] and [111] crystallographic orientations of diamond, respectively. The abscissa in the figure is the phonon frequency, the ordinate is the phonon lifetime, and the scale is the phonon density. The first row is a positive electric field, and the electric field strengths are 0.001 a.u., 0.002 a.u., 0.003 a.u. and 0.004 a.u. The second row is the negative electric field, and the electric field strengths are −0.001 a.u., −0.002 a.u., −0.003 a.u. and −0.004 a.u., respectively. It should be noted that the phonon mode density is calculated using the Gaussian KDE method, which is a method for estimating the probability density function of random variables in a non-parametric way (density of phonon modes in the frequency−lifetime plane. Its density is estimated using Gaussian−KDE. Estimation is a way to estimate the probability density function (PDF) of a random variable in a non-parametric way). In Figure 18, the diamond phonon lifetime density plots for the range of 0 a.u. consisting of red and cyan dots. At the same time, the red and cyan dots indicate that they do not coincide or coincide with the new density map, respectively. Taking 30 THz as the dividing point, the phonons on the left show a “fan-shaped” distribution, and the phonons on the right are relatively concentrated as a whole, resulting in the phonons between 30 and 40 THz clustering together with a very high density. After applying a positive electric field in the diamond [001] crystallographic direction, as shown in the first row in Figure 18, As the electric field strength increases, the “sector” part moves upward to the right as a whole, and the phonon lifetime becomes longer. When the electric field strength is 0.004 a.u., around 27 THz, the phonon lifetime is increased from 10 to 20 ps, and the phonon lifetime is increased by about 100%. Anyway, as the applied negative electric field becomes smaller and smaller, the “sector” part moves to the lower left as a whole, and the phonon lifetime also becomes shorter. When the electric field strength is −0.004 a.u., around 27 THz, the phonon lifetime decreases from 10 to 5 ps, and the phonon lifetime decreases by about 50%. However, regardless of the direction of the applied electric field, the shape of the phonon lifetime density distribution did not change significantly. The phonon lifetime density between 30 and 40 THz hardly shifts. The phonon distribution and phonon lifetime show almost the same phenomenon when positive and negative electric fields are applied to the [110] and [111] crystallographic orientations of the diamond unit cell. Only the electric field effect of the [111] crystallographic direction is more obvious, and the electric field effect of the [110] crystallographic direction is relatively less obvious. In summary, the application of positive and negative electric fields in the three crystallographic directions of the diamond unit cell cannot change the density distribution of phonons. The positive electric field will cause the phonon lifetime to become longer, and the negative electric field will cause the phonon lifetime to become shorter. This effect is mainly concentrated in phonons with frequencies less than 30 THz.

## 4. Conclusions

By applying an electric field to three crystallographic directions of diamond, the effect of the electric field on the thermal conductivity of the diamond lattice was studied by the first-principles calculation method. The response mechanism of the diamond lattice’s thermal transport to an electric field was revealed. The following conclusions are drawn:

(1) The response of diamond to the external electric field has anisotropy in three crystallographic directions. Moreover, the damage of positive and negative electric fields to the diamond phonon dispersion relationship is different. The three low-frequency acoustic branches are more sensitive to the electric field than the three high-frequency optical branches. The low-frequency lattice vibration of the diamond is more sensitive to the electric field.
(2) In the case of considering the electro-acoustic coupling, it is found by calculating the thermodynamic properties of a diamond under the electric field. The electric field will change the internal energy, free energy, entropy and specific heat capacity of the diamond. The [001] and [111] orientations of diamond are more sensitive to electric field than the [110] orientation. In the [111] crystallographic orientation, when the electric field strength is ±0.000736 a.u., the constant volume specific heat capacities are 1.45 × 10^−4^ eV/cell and 1.22 × 10^−4^ eV/cell, which are increased and decreased by 9.8% and −7.6%, respectively. The electric field causes changes in the extranuclear electron potential and kinetic energy of the C atoms in diamond, and the electroacoustic coupling causes the electrons to transfer part of the energy of the electric field to the phonons.
(3) The electric field will lead to a change in the thermal conductivity of the diamond lattice. Under the positive electric field, the thermal conductivity of the diamond lattice will increase, and conversely, the thermal conductivity of the diamond lattice will decrease under the negative electric field. When an electric field of ±0.004 a.u. is applied in the diamond [111] crystallographic direction and the temperature is 300 K, the thermal conductivity is 2654 $W\cdot m^{-1}K^{-1}$ and 1283 $W\cdot m^{-1}K^{-1}$. The corresponding percentages are 36.1% and −34%, respectively. The electric field will cause changes in the electron density distribution in diamond, destroying diamond lattice symmetry and leading to a change in the interatomic force constant. The average interatomic force increases linearly with the increase in the electric field strength.
(4) By calculating and analyzing the thermal transport properties of diamond phonons under the electric field, it is found that the electric field does not significantly change the phonon group velocity of the diamond. The response of diamond phonon thermal transport characteristics to electric field mainly focuses on the change of phonon mean free path, phonon–phonon interaction strength and phonon lifetime. Under a positive electric field, the increase in the mean free path of phonons, the enhancement of phonon–phonon interaction strength, and the prolongation of phonon lifetime all significantly promote the thermal conductivity of diamond lattice. On the contrary, under the negative electric field, the decrease in the phonon mean free path, the decrease in phonon–phonon interaction strength and the shortening of phonon lifetime significantly reduce the thermal conductivity of the diamond lattice. The response of the diamond phonon’s thermal transport to an electric field is mainly concentrated in the phonon part with a frequency of less than 30 THz.

## Figures and Tables

**Table 1:** The direction and magnitude of the electric field applied by the diamond when calculating the phonon dispersion curve.

Primitive Cell Base Vector	Electric Field Strength (Macroscopic Electric Field Unit: V∙cm^−1^, Microscopic Electric Field Unit: a.u.)			
$\left(11\bar{1}\right)$	±2.06 × 10^5^	±9.78 × 10^6^	±4.73 × 10^7^	±3.78 × 10^8^
$\left(\bar{1}\bar{1}1\right)$				
$\left(010\right)$				
$\left(0\bar{1}0\right)$	±4 × 10^−6^	±1.9 × 10^−5^	±9.2 × 10^−5^	±7.36 × 10^−4^
$\left(111\right)$				
$\left(\bar{1}\bar{1}\bar{1}\right)$				

Note: finite electric field atomic units: 1 a.u. = 514,220,624,373.482 V∙cm^−1^.

**Table 2:** Parameters of applied electric field in 3 crystallographic directions of diamond.

Crystal Orientation	Electric Field Strength (Macroscopic Electric Field Unit: V∙cm^−1^, Microscopic Electric Field Unit: a.u.)				
$\left[001\right]$	±5.14 × 10^7^	±5.14 × 10^8^	±1.03 × 10^9^	±1.54 × 10^9^	±2.06 × 10^9^
$$\left[00\bar{1}\right]$$					
$$\;\left[110\right]$$					
$\left[\bar{1}\bar{1}0\right]$	±1 × 10^4^	±1 × 10^3^	±2 × 10^3^	±3 × 10^3^	±4 × 10^3^
$$\left[111\right]$$					
$$\left[\bar{1}\bar{1}\bar{1}\right]$$					

**Table 3:** The rate of change of lattice thermal conductivity after applying an electric field in diamond [001], [110] and [111] crystal orientations. (*T* = 300 K).

**Crystal Orientation**	$-\Delta_{4}$	$-\Delta_{3}$	$-\Delta_{2}$	$-\Delta_{1}$	$\Delta_{1}$	$\Delta_{2}$	$\Delta_{3}\;$	$\Delta_{4}\;$
[001]	−125.808	−140.295	−151.474	−150.824	132.951	109.738	89.784	71.913
[110]	−95.855	−102.547	−105.554	−105.337	98.76	90.083	84.159	79.801
[111]	−157.797	−190.544	−226.948	−249.92	228.873	194.761	163.914	115.839

## Data Availability

The datasets generated during or analyzed during the current study are available from the corresponding author on reasonable request.

## References

[1] Larsson K. (2020). The Combined Influence of Dopant Species and Surface Termination on the Electronic Properties of Diamond Surfaces. C–J. Carbon Res..

[2] Silvestri L., Cervenka J., Prawer S., Ladouceur F. (2013). First Principle Study of Valence-Band Offsets at AlN/Diamond Heterojunctions. Diam. Relat. Mater..

[3] Rao L., Liu H., Shao W., Hu T., Xing X., Ren X., Zhou Y., Yang Q. (2020). Investigation on the Interface Characteristic between TiN and Diamond by First-Principles Calculation. Diam. Relat. Mater..

[4] Wei L., Kuo P.K., Thomas R.L., Anthony T.R., Banholzer W.F. (1993). Thermal Conductivity of Isotopically Modified Single Crystal Diamond. Phys. Rev. Lett..

[5] Twitchen D.J., Pickles C.S.J., Coe S.E., Sussmann R.S., Hall C.E. (2001). Thermal Conductivity Measurements on CVD Diamond. Diam. Relat. Mater..

[6] Hartmann J., Voigt P., Reichling M. (1997). Measuring Local Thermal Conductivity in Polycrystalline Diamond with a High Resolution Photothermal Microscope. J. Appl. Phys..

[7] Chakraborty P., Xiong G., Cao L., Wang Y. (2018). Lattice Thermal Transport in Superhard Hexagonal Diamond and Wurtzite Boron Nitride: A Comparative Study with Cubic Diamond and Cubic Boron Nitride. Carbon.

[8] Ciupiński Ł., Kruszewski M.J., Grzonka J., Chmielewski M., Zielińsk R., Moszczyńska D., Michalski A. (2017). Design of Interfacial Cr_3_C_2_ Carbide Layer via Optimization of Sintering Parameters Used to Fabricate Copper/Diamond Composites for Thermal Management Applications. Mater. Des..

[9] Bai G., Zhang Y., Dai J., Wang L., Wang X., Wang J., Kim M.J., Chen X., Zhang H. (2019). Tunable Coef Fi Cient of Thermal Expansion of Cu-B/Diamond Composites Prepared by Gas Pressure in Fi Ltration. J. Alloys Compd..

[10] Arai S., Ueda M. (2020). Fabrication of High Thermal Conductivity Copper/Diamond Composites by Electrodeposition under Potentiostatic Conditions. J. Appl. Electrochem..

[11] Liu Z., Zheng S., Lu Z., Pu J., Zhang G. (2018). Adhesive Transfer at Copper/Diamond Interface and Adhesion Reduction Mechanism with Fluorine Passivation: A First-Principles Study. Carbon.

[12] Ren S., Shen X., Guo C., Liu N., Zang J., He X., Qu X. (2011). Effect of Coating on the Microstructure and Thermal Conductivities of Diamond-Cu Composites Prepared by Powder Metallurgy. Compos. Sci. Technol..

[13] Mortazavi B., Shojaei F., Zhuang X., Pereira L.F.C. (2021). First-Principles Investigation of Electronic, Optical, Mechanical and Heat Transport Properties of Pentadiamond: A Comparison with Diamond. Carbon Trends.

[14] Barman S., Srivastava G.P. (2006). Quantitative Estimate of Phonon Scattering Rates in Different Forms of Diamond. Phys. Rev. B.

[15] Vasil’ev N.S., Gorelik V.S. (2011). Properties of Optical and Acoustic Phonons in Diamond Crystals. Bull. Lebedev. Phys. Inst..

[16] Lu T., Chen Q. (2021). Ultrastrong Regulation Effect of the Electric Field on the All-Carboatomic Ring Cyclo [18]Carbon**. ChemPhysChem.

[17] Ran Z., Du B., Li J., Liang H., Kong X., Jiang J., Wang M. (2019). Electric Field Regulation of Insulator Interface by FGM With Conductivity for Superconducting-GIL. IEEE Trans. Appl. Supercond..

[18] Duan Y.-J., Zhao Y., Cheng S.-B., Wei Q. (2022). On the Precise and Continuous Regulation of the Superatomic and Spectroscopic Behaviors of the Quasi-Cubic W_4_C_4_ Cluster by the Oriented External Electric Field. J. Phys. Chem. A.

[19] Souza I., Íñiguez J., Vanderbilt D. (2002). First-Principles Approach to Insulators in Finite Electric Fields. Phys. Rev. Lett..

[20] Kim K., Ju H., Kim J. (2016). Filler Orientation of Boron Nitride Composite via External Electric Field for Thermal Conductivity Enhancement. Ceram. Int..

[21] Huber W.H., Hernandez L.M., Goldman A.M. (2000). Electric Field Dependence of the Thermal Conductivity of Quantum Paraelectrics. Phys. Rev. B.

[22] Aikawa Y., Baba K., Shohata N., Yoneda H., Ueda K. (1996). Photoconductive Properties of Polycrystalline Diamond under High Electric Field Strength. Diam. Relat. Mater..

[23] Sankara Reddy K.S., Satyam M. (1995). Structural Ordering of Diamond like Carbon Films by Applied Electric Field. Solid State Commun..

[24] Gao Y., Okada S. (2022). Electronic Properties of Diamond Nanowires under an External Electric Field. Diam. Relat. Mater..

[25] Lambert N., Taylor A., Hubík P., Bulíř J., More-Chevalier J., Karaca H., Fleury C., Voves J., Šobáň Z., Pogany D. (2020). Modeling Current Transport in Boron-Doped Diamond at High Electric Fields Including Self-Heating Effect. Diam. Relat. Mater..

[26] Gonze X., Amadon B., Antonius G., Arnardi F., Baguet L., Beuken J.-M., Bieder J., Bottin F., Bouchet J., Bousquet E. (2020). The Abinitproject: Impact, Environment and Recent Developments. Comput. Phys. Commun..

[27] Romero A.H., Allan D.C., Amadon B., Antonius G., Applencourt T., Baguet L., Bieder J., Bottin F., Bouchet J., Bousquet E. (2020). ABINIT: Overview and Focus on Selected Capabilities. J. Chem. Phys..

[28] Gonze X., Jollet F., Abreu Araujo F., Adams D., Amadon B., Applencourt T., Audouze C., Beuken J.-M., Bieder J., Bokhanchuk A. (2016). Recent Developments in the ABINIT Software Package. Comput. Phys. Commun..

[29] Gonze X., Amadon B., Anglade P.-M., Beuken J.-M., Bottin F., Boulanger P., Bruneval F., Caliste D., Caracas R., Côté M. (2009). ABINIT: First-Principles Approach to Material and Nanosystem Properties. Comput. Phys. Commun..

[30] Torrent M., Jollet F., Bottin F., Zérah G., Gonze X. (2008). Implementation of the Projector Augmented-Wave Method in the ABINIT Code: Application to the Study of Iron under Pressure. Comput. Mater. Sci..

[31] Togo A., Chaput L., Tanaka I. (2015). Distributions of Phonon Lifetimes in Brillouin Zones. Phys. Rev. B.

[32] Chaput L. (2013). Direct Solution to the Linearized Phonon Boltzmann Equation. Phys. Rev. Lett..

[33] Momma K., Izumi F. (2011). VESTA 3 for Three-Dimensional Visualization of Crystal, Volumetric and Morphology Data. J. Appl. Crystallogr..

[34] Petretto G., Gonze X., Hautier G., Rignanese G.-M. (2018). Convergence and Pitfalls of Density Functional Perturbation Theory Phonons Calculations from a High-Throughput Perspective. Comput. Mater. Sci..

[35] Brunin G., Miranda H.P.C., Giantomassi M., Royo M., Stengel M., Verstraete M.J., Gonze X., Rignanese G.-M., Hautier G. (2020). Phonon-Limited Electron Mobility in Si, GaAs, and GaP with Exact Treatment of Dynamical Quadrupoles. Phys. Rev. B.

[36] Zhao Y., Yan F., An Y. (2022). Formation and Performance of Diamond (111)/Cu Interface from First-Principles Calculation. Coatings.

[37] Park M., Choi W.B., Schlesser R., Sowers A.T., Bergman L., Nemanich R.J., Sitar Z., Hren J.J., Cuomo J.J. (1998). The Effect of Substitutional Nitrogen Incorporation on Electron Emission from CVD Diamond. Proceedings of the Eleventh International Vacuum Microelectronics Conference, IVMC’98 (Cat. No. 98TH8382).

[38] Irie M., Endo S., Wang C.L., Ito T. (2003). Fabrication and Properties of Lateral P-i-p Structures Using Single-Crystalline CVD Diamond Layers for High Electric Field Applications. Diam. Relat. Mater..

[39] Giustino F. (2017). Electron-Phonon Interactions from First Principles. Rev. Mod. Phys..

[40] Abrikosov A.A., Gorkov L.P., Dzyaloshinskii E. (1975). Methods of Quantum Field Theory in Statistical Physics.

[41] Poncé S., Li W., Reichardt S., Giustino F. (2020). First-Principles Calculations of Charge Carrier Mobility and Conductivity in Bulk Semiconductors and Two-Dimensional Materials. Reports Prog. Phys..

[42] Madsen G.K.H., Carrete J., Verstraete M.J. (2018). BoltzTraP2, a Program for Interpolating Band Structures and Calculating Semi-Classical Transport Coefficients. Comput. Phys. Commun..

[43] Nunes R.W., Gonze X. (2001). Berry-Phase Treatment of the Homogeneous Electric Field Perturbation in Insulators. Phys. Rev. B.

[44] Resta R. (1994). Macroscopic Polarization in Crystalline Dielectrics: The Geometric Phase Approach. Rev. Mod. Phys..

[45] Tang D.S., Cao B.Y. (2021). Phonon Thermal Transport Properties of GaN with Symmetry-Breaking and Lattice Deformation Induced by the Electric Field. Int. J. Heat Mass Transf..

[46] Qi R., Li N., Du J., Shi R., Huang Y., Yang X., Liu L., Xu Z., Dai Q., Yu D. (2021). Four-Dimensional Vibrational Spectroscopy for Nanoscale Mapping of Phonon Dispersion in BN Nanotubes. Nat. Commun..

[47] De Leonardis F., Soref R.A., Passaro V.M.N. (2017). Dispersion of Nonresonant Third-Order Nonlinearities in Silicon Carbide. Sci. Rep..

[48] Calzolari A., Nardelli M.B. (2013). Dielectric Properties and Raman Spectra of ZnO from a First Principles Finite-Differences/Finite-Fields Approach. Sci. Rep..

[49] Chen L., Chen S., Hou Y. (2019). Understanding the Thermal Conductivity of Diamond/Copper Composite by First-Principles Calculations. Carbon.

[50] Kim H., Vogelgesang R., Ramdas A., Rodriguez S., Grimsditch M. (1998). Electronic Raman and Infrared Spectra of Acceptors in Isotopically Controlled Diamonds. Phys. Rev. B-Condens. Matter Mater. Phys..

[51] Sparavigna A. (2002). Influence of Isotope Scattering on the Thermal Conductivity of Diamond. Phys. Rev. B-Condens. Matter Mater. Phys..

